# Repurposing Cancer Drugs Batimastat and Marimastat to Inhibit the Activity of a Group I Metalloprotease from the Venom of the Western Diamondback Rattlesnake, *Crotalus atrox*

**DOI:** 10.3390/toxins12050309

**Published:** 2020-05-09

**Authors:** Harry J. Layfield, Harry F. Williams, Divyashree Ravishankar, Amita Mehmi, Medha Sonavane, Anika Salim, Rajendran Vaiyapuri, Karthik Lakshminarayanan, Thomas M. Vallance, Andrew B. Bicknell, Steven A. Trim, Ketan Patel, Sakthivel Vaiyapuri

**Affiliations:** 1 School of Pharmacy, University of Reading, Reading RG6 6UB, UK; harrylayfield@gmail.com (H.J.L.); harry@toxiven.com (H.F.W.); divyasri.april86@gmail.com (D.R.); A.Mehmi@student.reading.ac.uk (A.M.); m.sonavane@pgr.reading.ac.uk (M.S.); anika.salim@pgr.reading.ac.uk (A.S.); T.M.Vallance@pgr.reading.ac.uk (T.M.V.); 2 Toxiven Biotech Private Limited, Coimbatore, Tamil Nadu 641042, India; raj@toxiven.com (R.V.); karthik@toxiven.com (K.L.); 3 School of Biological Sciences, University of Reading, Reading RG6 6UB, UK; a.b.bicknell@reading.ac.uk (A.B.B.); ketan.patel@reading.ac.uk (K.P.); 4 Venomtech Limited, Sandwich, Kent CT13 9ND, UK; s.trim@venomtech.co.uk

**Keywords:** *Crotalus atrox*, metalloprotease, snake venom, neglected tropical disease, rattlesnake, batimastat, marimastat, antivenom

## Abstract

Snakebite envenomation causes over 140,000 deaths every year, predominantly in developing countries. As a result, it is one of the most lethal neglected tropical diseases. It is associated with incredibly complex pathophysiology due to the vast number of unique toxins/proteins present in the venoms of diverse snake species found worldwide. Here, we report the purification and functional characteristics of a Group I (PI) metalloprotease (CAMP-2) from the venom of the western diamondback rattlesnake, *Crotalus atrox*. Its sensitivity to matrix metalloprotease inhibitors (batimastat and marimastat) was established using specific in vitro experiments and in silico molecular docking analysis. CAMP-2 shows high sequence homology to atroxase from the venom of *Crotalus atrox* and exhibits collagenolytic, fibrinogenolytic and mild haemolytic activities. It exerts a mild inhibitory effect on agonist-induced platelet aggregation in the absence of plasma proteins. Its collagenolytic activity is completely inhibited by batimastat and marimastat. Zinc chloride also inhibits the collagenolytic activity of CAMP-2 by around 75% at 50 μM, while it is partially potentiated by calcium chloride. Molecular docking studies have demonstrated that batimastat and marimastat are able to bind strongly to the active site residues of CAMP-2. This study demonstrates the impact of matrix metalloprotease inhibitors in the modulation of a purified, Group I metalloprotease activities in comparison to the whole venom. By improving our understanding of snake venom metalloproteases and their sensitivity to small molecule inhibitors, we can begin to develop novel and improved treatment strategies for snakebites.

## 1. Introduction

In 2017, snakebite envenomation (SBE) was reinstated to the list of neglected tropical diseases by the World Health Organisation [1,2]. SBE is estimated to occur in at least 1.8–2.7 million people, resulting in around 80,000–137,000 deaths and over 400,000 amputations worldwide per year [3]. The distribution of fatalities is primarily concentrated in rural tropical areas that are some of the world’s poorest and most healthcare deprived communities [1,4,5]. Prompt access to antivenom therapy and appropriate medical facilities is crucial in order to protect victims from death, potential extensive limb injuries and the possibility of subsequent, long-term disabilities [4]. The current antivenoms are considered to be sub-optimal in preventing venom-induced tissue damage due to their inability to access the affected local tissues [4,5]. Hence, the development of small molecules that are able to neutralise the locally acting venom components would be highly beneficial in treating SBE, specifically SBE-induced muscle damage and/or tissue necrosis.

Snake venoms are a complex mixture of bioactive proteins and peptides that have evolved over time to assist in subduing and killing prey as quickly as possible, as well as having a secondary role in prey digestion and defence [5]. The clinical effects of SBE range from mild local reactions to more serious life-threatening conditions depending on a variety of variables including the size, species and locality of the snake [6,7], ontogeny [8,9], body mass and health of the victim and the total volume of venom injected [1,10]. Snake venoms are composed of both enzymatic and non-enzymatic components. The enzymatic components of viper venoms primarily include the snake venom metalloproteases (SVMPs), serine proteases and phospholipase A_2_ (PLA_2_), whereas non-enzymatic venom components include three-finger toxins, C-type lectins and disintegrins amongst many others [5]. The western diamondback rattlesnake, *Crotalus atrox* (*C. atrox*) is likely to be responsible for the majority of SBE-induced fatalities in Northern Mexico [11]. *C. atrox* venom has an abundance of two major protein families, SVMPs and serine proteases, which together account for approximately 70% of the total protein found within the venom [12].

SVMPs are zinc-dependent enzymes that vary in molecular mass from approximately 20 to 100 kDa and are responsible for the haemorrhagic effects and local tissue damage frequently seen upon viper envenomation [13,14]. SVMPs are classified into PI to PIV depending on the presence of additional domains [15]: PI—only a metalloprotease domain; PII—a metalloprotease and a disintegrin domain; PIII—a metalloprotease domain, a disintegrin-like and a cysteine-rich domain; PIV—two C-type lectin domains in addition to all the domains present in PIII. SVMPs are involved in a wide range of toxic activities, including the degradation of collagen and other basement membrane components, fibrinogen and a range of other proteins [13]. The peptidomimetic molecules, batimastat and marimastat are broad-spectrum matrix metalloprotease (MMPs) inhibitors [16] that have been proposed as next generation treatment options for the SVMP-induced effects of SBE [17]. This inhibition is achieved by mimicking the cleavage site of natural substrates and binding to the zinc ion found in the active site of these proteases. In this way, batimastat and the orally bioavailable and similar compound, marimastat are able to inhibit both matrix metalloproteases as well as SVMPs [5]. An improved understanding of MMPs, their inhibitors, and their relationship with SVMPs will aid in the development of improved therapeutic strategies for SBE. 

SVMPs in *C. atrox* venom account for 49.7% of total venom, which breaks down further to 22.4% PI and 27.3% PIII SVMPs [12]. In order to determine the therapeutic potential of batimastat and marimastat against PI venom metalloproteases, here, we report the purification and functional characterisation of a PI metalloprotease with a molecular weight of around 23 kDa from the venom of *C. atrox*. The sensitivity of the purified protein to inhibition by batimastat and marimastat was established in comparison to the whole *C. atrox* venom. Together, this study supports the potential beneficial effects of these molecules against the broad spectrum of pathological effects induced by SVMPs.

## 2. Results

### 2.1. Purification and Identification of CAMP-2

**Figure 1:**
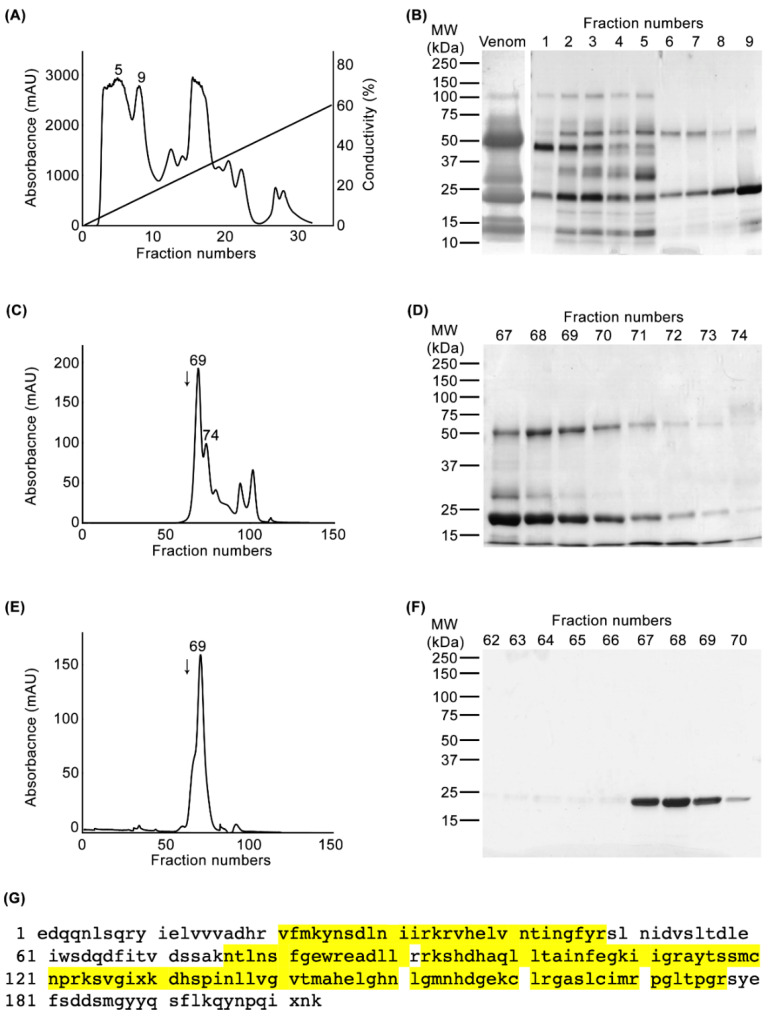
Purification and identification of CAMP-2. 50 mg of whole *C. atrox* venom was fractionated using a cation exchange chromatography column (**A**) And the collected fractions were analysed by SDS-PAGE (**B**). (**C**) A chromatogram showing the gel filtration chromatography profile of fractions 6 to 9 collected from the cation exchange column. (**D**) SDS-PAGE analysis of selected fractions resulting from the gel filtration chromatography. (**E**) The chromatogram from the second run of gel filtration chromatography using fractions 67–72 from the previous run, and SDS-PAGE analysis showing the purified protein (**F**). The gels shown were stained with Coomassie brilliant blue. The arrow in (**C**) and (**E**) indicates the position of chymotrypsinogen A (25 kDa), which was used as a molecular weight marker in the same gel filtration column. (**G**) Tryptic digested peptides of the purified protein were analysed by mass spectrometry and the data show 52.2% identity to a previously sequenced PI SVMP, atroxase, from *C. atrox* venom. The mass spectrometry-identified peptide sequences of purified protein are shown in yellow on the sequence of atroxase.

To purify a PI SVMP from the venom of *C. atrox*, we deployed a two-dimensional chromatography approach. Initially, 50 mg of whole *C. atrox* venom was applied to a cation-exchange (SP-HP) chromatography column (Figure 1A) followed by the analysis of collected fractions using SDS-PAGE (Figure 1B). Due to the abundance of the target protein at a molecular weight of around 23 kDa (typical for a PI SVMP [18]), the selected fractions (6–9) were further fractionated by gel filtration (Superdex 75, 1.6 × 70 cm) chromatography (Figure 1C). Following SDS-PAGE analysis (Figure 1D), selected fractions (67–72) were further run through the same gel filtration column to remove any impurities from the protein of interest (Figure 1E,F). Finally, a pure protein with a molecular weight of approximately 23 kDa was isolated, which we henceforth refer to as CAMP-2 (denoting the second SVMP that we have isolated from the venom of *C. atrox*). The molecular weight of the isolated protein was confirmed under native conditions using chymotrypsinogen A (25 kDa) as a marker in gel filtration chromatography (indicated with an arrow in Figure 1C,E) and under denaturing (reduced) conditions using SDS-PAGE (Figure 1F). 

To determine the identity of the purified protein, it was subjected to trypsin digestion and subsequent analysis by mass spectrometry (Figure 1G). Mascot analysis of the MS/MS data suggested that the isolated protein possesses a high sequence identity to atroxase, a 23 kDa SVMP from the venom of *C. atrox* [19,20]. The peptide sequences resulting from the mass spectrometry covered 52.2% of atroxase, suggesting that the purified protein is highly likely to be atroxase although we cannot confirm this due to the lack of complete sequencing from this study. However, these data confirm that the purified protein is a PI SVMP with a molecular weight of 23 kDa, and it is likely to be atroxase, which was previously purified and characterised as a non-haemorrhagic protease with fibrin(ogen)olytic activities [20,21,22,23]. 

### 2.2. CAMP-2 Exerts Collagenolytic, Fibrinogenolytic and Haemolytic Activities

**Figure 2:**
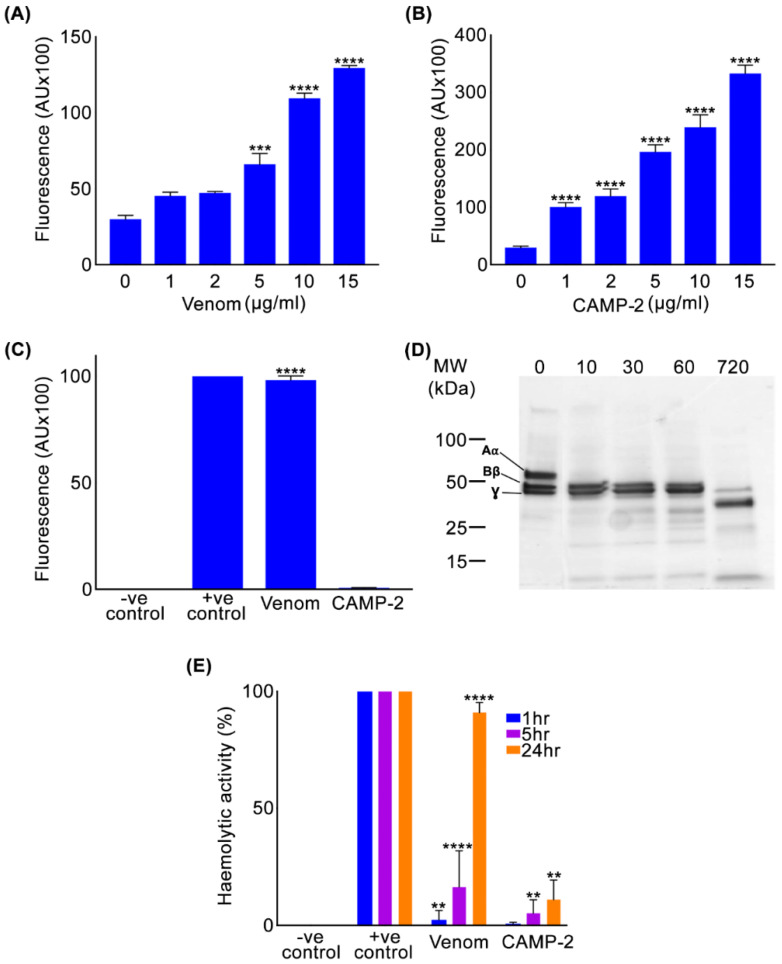
Functional characterisation of CAMP-2. The metalloprotease (collagenolytic) activity of different concentrations of whole *C. atrox* venom (**A**) and CAMP-2 (**B**) was assessed using DQ gelatin (a fluorogenic substrate). (**C**) the PLA_2_ activity of whole *C. atrox* venom and CAMP-2 was analysed using EnzCheck^TM^ lipid-based substrate. (**D**) The fibrinogenolytic activity of CAMP-2 was analysed by incubating it with human fibrinogen and the samples collected at different time points (0–720 min as indicated at the top) were assessed by SDS-PAGE and Coomassie staining. (**E**) Haemolytic activity of whole *C. atrox* venom and CAMP-2 was analysed by incubating them with human red blood cells and analysing the cell-free supernatant by spectrometry. The positive (+ve) control represents the complete lysis achieved using 1% (*v*/*v*) Triton-X in PBS. Data represent mean ± S.D. (*n* = 3). The *p-*values shown were calculated using one-way ANOVA followed by posthoc Tukey’s test using GraphPad Prism (** *p* ≤ 0.01, *** *p* ≤ 0.001 and **** *p* ≤ 0.0001).

To determine the roles of CAMP-2, various functional assays using synthetic and natural substrates were performed in comparison to the whole *C. atrox* venom. Fluorogenic substrates such as DQ-gelatin and EnzCheck^TM^ lipid-based substrate were used to assess if CAMP-2 possesses metalloprotease (collagenolytic) and PLA_2_ activities, respectively. Similar to the whole *C. atrox* venom (Figure 2A), CAMP-2 (Figure 2B) exhibited strong collagenolytic activity. While the whole *C. atrox* venom showed clear PLA_2_ activity, CAMP-2 did not display any PLA_2_ activity (Figure 2C). These data not only suggest that CAMP-2 is an SVMP, but it is also free from any PLA_2_ impurities which have a similar molecular weight. Moreover, human fibrinogen, a plasma protein which is a natural substrate for some SVMPs, was incubated with CAMP-2, before the digest was analysed by SDS-PAGE to determine its effects on fibrinogen. This analysis showed that CAMP-2 has fibrinogenolytic activity and notably, it exerts high specificity for the Aα chain of fibrinogen, as it was completely digested within 10 min (Figure 2D) although over a longer time (e.g., 12 h), it also began to degrade the Bβ chain while the γ chain of fibrinogen remained largely unaffected. Similarly, CAMP-2 showed a mild haemolytic effect compared to the whole venom when incubated with human red blood cells over 24 h (Figure 2E). These data suggest that CAMP-2 is a collagenolytic, fibrinogenolytic and mildly haemolytic enzyme.

### 2.3. CAMP-2 Inhibits Human Platelet Aggregation

**Figure 3:**
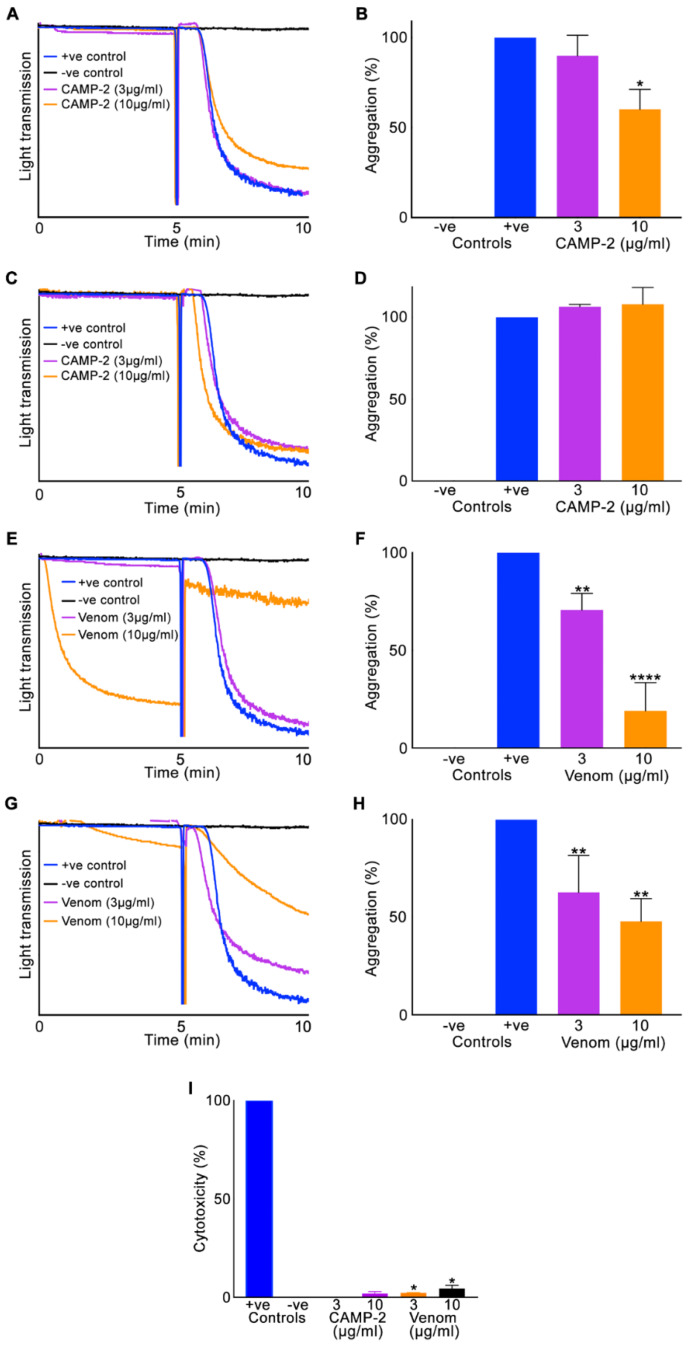
Effect of CAMP-2 on human platelets. The effect of CAMP-2 on human isolated platelets (**A**,**B**) and platelet-rich plasma (PRP) (**C**,**D**) was analysed in the presence and absence of a platelet agonist, cross-linked collagen-related peptide (CRP-XL) by aggregometry. Similar experiments using isolated platelets (**E**,**F**) and PRP (**G**,**H**) were performed using the whole *C. atrox* venom. The aggregation traces shown are representative of three separate experiments. (**I**) The cytotoxicity of CAMP-2 and the whole venom was determined by incubating them with human platelets for 30 min and analysing using a lactate dehydrogenase (LDH) assay kit by spectrometry. The positive (+ve) control (100% cytotoxicity) was achieved using a lysis buffer provided in the kit. Data represent mean ± S.D. (*n* = 3). The *p*-values shown were calculated using one-way ANOVA followed by a posthoc Tukey’s test using GraphPad Prism (* *p* ≤ 0.05, ** *p* ≤ 0.01, and **** *p* ≤ 0.0001).

To determine if CAMP-2 is able to affect human platelet function, platelet aggregation assays using platelet-rich plasma (PRP) and isolated platelets from human whole blood were used. CAMP-2 at both low (3 μg/mL) and high (10 μg/mL) concentrations did not induce platelet aggregation on its own (indicated as 0–5 min in aggregation traces shown in Figure 3). However, 10 μg/mL CAMP-2 inhibited (Figure 3A,B) platelet aggregation (by around 25%) induced by a cross-linked collagen-related peptide (CRP-XL) when isolated platelets were used, although the low concentration did not show any significant effect. This inhibitory effect was absent when PRP (i.e., in the presence of plasma proteins) was used (Figure 3C,D). When the whole *C. atrox* venom was used, at a low concentration (3 μg/mL) it displayed mild inhibitory effects on CRP-XL induced platelet aggregation, although at a higher (10 μg/mL) concentration it has possibly lysed (even in the absence of an agonist as shown in the aggregation traces between 0 and 5 minutes) the platelets when both isolated platelets (Figure 3E,F) and PRP (Figure 3G,H) were used. These results demonstrate that although CAMP-2 is able to display a minimal inhibitory effect on platelet aggregation when isolated platelets were used, it is unable to affect their function in the presence of plasma proteins, indicating that this may not be its primary role in humans. However, the whole venom may induce the lysis of platelets based on the concentrations injected during the bite.

In order to assess if CAMP-2 and the whole venom are able to exert direct cytotoxic effects on platelets, lactate dehydrogenase (LDH) assay using human isolated platelets was performed. These results indicated that CAMP-2 does not have any cytotoxic effects on platelets at the concentrations tested in this study, although the whole venom displayed a mild (around 10% at 10 μg/mL) cytotoxic effect on platelets (Figure 3I) similar to its effect on platelet aggregation (Figure 3E–H). 

### 2.4. Chlorides and a Metal Chelator Affect Metalloprotease Activity of CAMP-2

**Figure 4:**
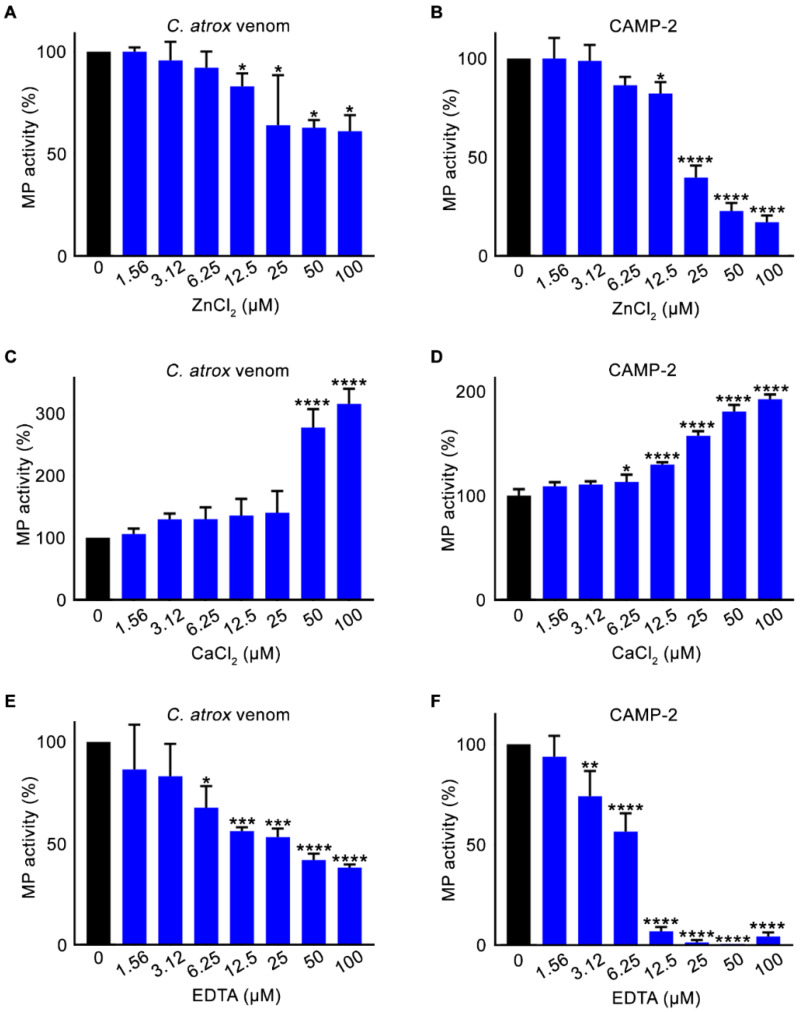
The effect of chlorides and a metal chelator on the metalloprotease activity of CAMP-2. The effect of different concentrations of zinc chloride on the metalloprotease (collagenolytic) activity of whole venom (**A**) and CAMP-2 (**B**) was analysed using DQ-gelatin as a substrate by spectrofluorimetry. Similarly, the effect of various concentrations of calcium chloride (**C**,**D**) and a metal chelator, EDTA (**E**,**F**) on whole *C. atrox* venom, as well as on CAMP-2, was analysed. Data represent mean ± S.D. (*n* = 3). The *p*-values shown are as calculated by one-way ANOVA followed by posthoc Tukey’s test using GraphPad Prism (* *p* ≤ 0.05, ** *p* ≤ 0.01, *** *p* ≤ 0.001 and **** *p* ≤ 0.0001).

As zinc-dependent proteases, SVMPs rely on free divalent cations such as calcium for catalysis, the impact of various metal chlorides on the metalloprotease activity of CAMP-2 was assessed. Diverse concentrations of both zinc and calcium chloride were used to assess if they would interfere with the metalloprotease (collagenolytic) activity of CAMP-2 and the whole *C. atrox* venom. The results demonstrate that while zinc chloride significantly reduced the metalloprotease activity observed with both the whole venom (Figure 4A) and CAMP-2 (Figure 4B), calcium chloride potentiated this activity of whole venom (Figure 4C) and CAMP-2 (Figure 4D). 

Similarly, the effect of a metal chelator on proteolytic activity was assessed using ethylenediaminetetraacetic acid (EDTA). The metalloprotease activity of both whole venom (Figure 4E) and CAMP-2 (Figure 4F) was strongly inhibited by different concentrations of EDTA, further corroborating that CAMP-2 is a metalloprotease and sensitive to metal chelators such as EDTA.

### 2.5. Marimastat and Batimastat Inhibit the Activity of CAMP-2

**Figure 5:**
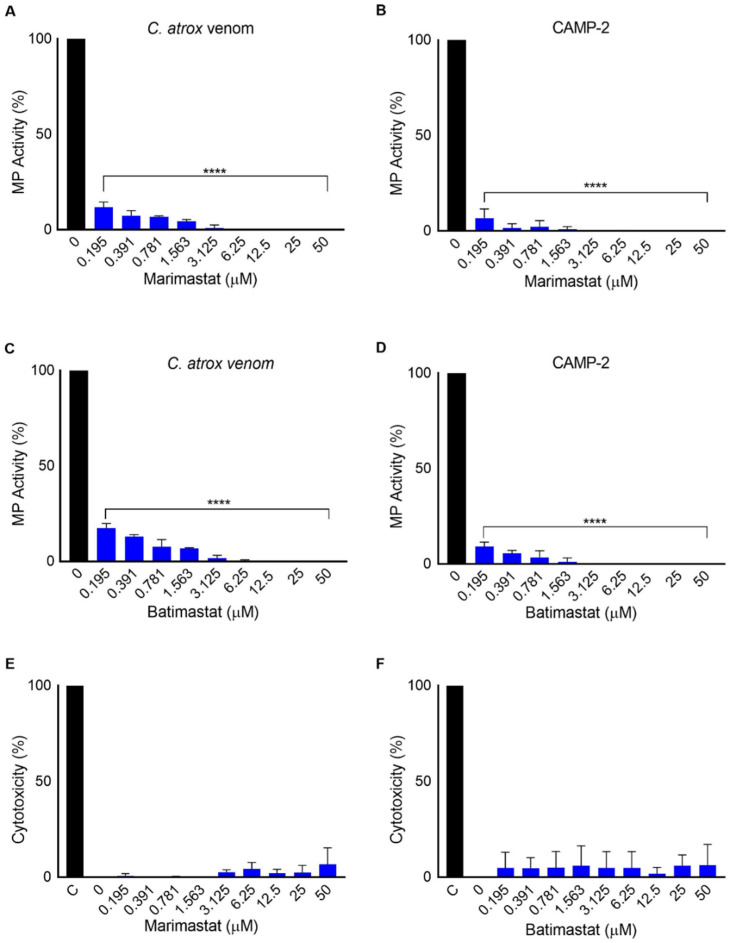
Inhibitory effects of marimastat and batimastat on metalloprotease activity of CAMP-2 and whole *C. atrox* venom. The inhibitory effects of various concentrations of marimastat (on whole *C. atrox* venom (**A**) or CAMP-2 (**B**)) and batimastat (on whole *C. atrox* venom (**C**) or CAMP-2 (**D**)) were quantified in vitro using a fluorogenic substrate, DQ-gelatin by spectrofluorimetry. The inhibitors were incubated with the whole venom or CAMP-2 for 5 min prior to the addition of DQ-gelatin and further incubation of 30 min prior to measuring the level of fluorescence by spectrofluorimetry. The cytotoxicity of marimastat (**E**) and batimastat (**F**) on human platelets was also assessed using a lactate dehydrogenase (LDH) assay. C represents the positive control (100% lysis) achieved using the lysis buffer provided in the kit. Data represent mean ± S.D. (*n* = 3). The *p*-values shown are as calculated by one-way ANOVA followed by posthoc Tukey’s test using GraphPad Prism (**** *p* ≤ 0.0001).

After confirming the biological actions of CAMP-2, the effects of MMP inhibitors, batimastat and marimastat on this protein in comparison to the whole *C. atrox* venom were analysed. Different concentrations of these inhibitors were incubated with 2 μg of CAMP-2 or whole venom for 5 min prior to analysing their metalloprotease (collagenolytic) activity using DQ-gelatin by spectrofluorimetry. The results suggest that marimastat (Figure 5A,B) and batimastat (Figure 5C,D) are able to inhibit the metalloprotease activity of both the whole venom and CAMP-2. A concentration of around 3 μM was able to completely inhibit the activity of both the whole venom and CAMP-2. Moreover, neither marimastat nor batimastat exerted any cytotoxic effects on human platelets, as analysed by an LDH assay (Figure 5E,F). These data suggest that these two MMP inhibitors are effective at inhibiting PI SVMPs such as CAMP-2, and they do not possess any cytotoxic activities.

### 2.6. Interactions of CAMP-2 with Batimastat and Marimastat

The mass spectrometry (Figure 1G) and functional data suggested that CAMP-2 is highly likely to be a previously characterised PI SVMP, atroxase, from the venom of *C. atrox*. Hence, we have used the complete sequence of atroxase (Uniprot accession number: Q91401) as CAMP-2 in this study for further analysis. Sequence analysis confirmed that CAMP-2 displayed approximately 74% and 50% identity with other metalloproteases, atrolysin C (PI SVMP; PDB accession numbers: 1DTH and 1ATL) and catrocollastatin (PIII SVMP; PDB accession number: 2DW0), respectively, from the same *C. atrox* venom. A three-dimensional structure of CAMP-2 was generated using homology modeling in Swiss Model Server [24] based on the template of adamalysin II (PDB accession number: 4AIG) from the venom of *Crotalus adamanteus*. A pairwise sequence alignment confirmed that CAMP-2 exhibited 83% identity with adamalysin II and, therefore, this protein has been used as a template instead of atrolysin C to develop the structure of CAMP-2. The superimposing of CAMP-2 with the structure of adamalysin has displayed a minimum rmsd of 0.142 Å, which further emphasises the reliability of this model.

**Figure 6:**
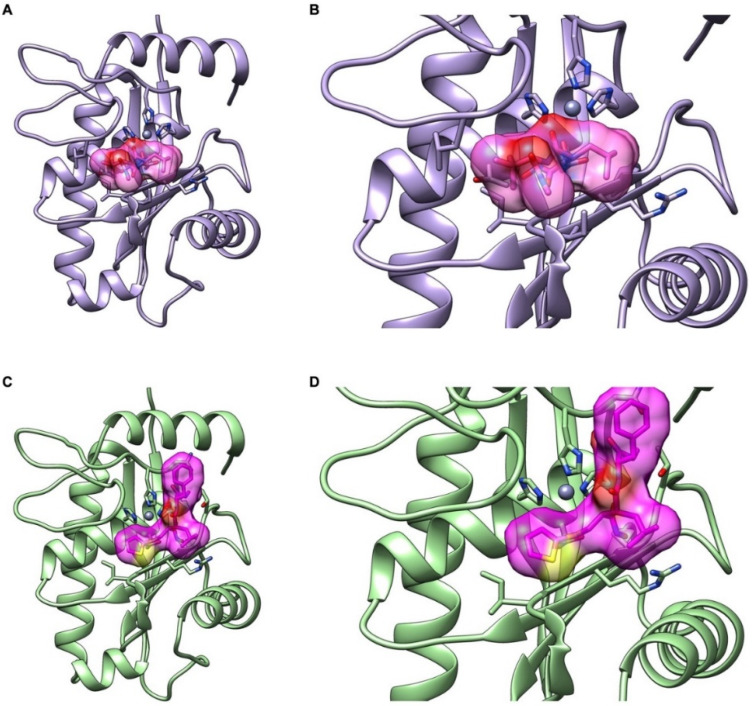
Interactions of marimastat and batimastat with the modeled structure of CAMP-2. The structure of CAMP-2 was modeled using the crystal structure of adamalysin II, a highly (83%) similar SVMP from the venom of *Crotalus adamanteus* as a template. This structure was used for molecular docking analysis (using AutoDock) to evaluate the interactions of marimastat (**A**,**B**) and batimastat (**C**,**D**) with CAMP-2. The structures shown in (**B**,**D**) are enlarged views of the docking complex (catalytic site with the small molecule inhibitors batimastat and marimastat).

Following the validation of the modeled structure of CAMP-2, molecular docking analysis was performed using AutoDock 4.2 [25] with the chemical structures (obtained from PubChem) of batimastat and marimastat. Three-dimensional atomic coordinates of these compounds were generated using the Online SMILES Translator and Structure File Generator (NCI NIH server (https://cactus.nci.nih.gov/translate/)). Subsequently, the compounds were subjected to energy-minimization using the PRODRG server. The docking analysis revealed that both the inhibitors exhibited comparable binding energy and reliable hydrogen bond interactions with the active site residues of CAMP-2 (Table 1). While batimastat possessed slightly higher binding energy and inhibitory constant, marimastat formed a greater number of hydrogen bonds and hydrophobic interactions with CAMP-2 (Figure 6A–D). Notably, batimastat was found to interact with one of the catalytic triad residues, His 154, through its backbone nitrogen, while marimastat formed a bifurcated hydrogen bond interaction with the active site, Glu 145, through its sidechain oxygen (Table 1). These results are in line with the inhibitory effects observed in the metalloprotease assay (Figure 5A–D).

## 3. Discussion

Common effects of SBE caused by vipers include haemorrhage, rhabdomyolysis, oedema and severe muscle damage, and often these result in permanent disabilities [1]. These consequences can trigger serious lifestyle and socio-economic ramifications for victims, particularly those in rural areas with difficulty in accessing affordable and effective antivenom [26,27]. The most abundant proteins within viper venoms that are responsible for many of these consequences are SVMPs [13]. The currently used antivenom therapy has proven to be largely ineffective in treating local tissue damage induced by SVMPs due to their inability to access the damaged site because of the large size of antibodies and damaged/blocked blood capillaries around the bite site [5]. As a result, clinicians are forced to employ surgical procedures such as fasciotomy, debridement or limb amputation in severe cases to treat/remove the affected tissues [27]. Therefore, developing an alternative therapy is critical to prevent/treat SBE-induced muscle damage and subsequent disabilities. The use of MMP inhibitors such as batimastat and marimastat has been proposed to inhibit the activities of SVMPs from various venoms [5]. These drugs were originally developed for cancer but failed in clinical trials—batimastat due to its poor solubility and low oral bioavailability, and marimastat, although it showed much promise and reached phase II and III clinical trials, was eventually discontinued after failing to demonstrate a survival benefit [28]. Its longer-term use also leads to debilitating “musculoskeletal syndrome” [29]. Despite these failings, their applications (particularly, orally bioavailable marimastat) in treating the acute local effects induced by SBE would be greatly beneficial to prevent SBE-induced disabilities. Hence, in this study, we have evaluated the efficacy of batimastat and marimastat, specifically on a purified PI SVMP from the venom of *C. atrox*. 

We have previously used the two-dimensional chromatography approach (e.g., a combination of ion exchange and gel filtration) as an effective method to purify various venom components, including a 50 kDa PIII SVMP CAMP from the venom of *C. atrox* [14,30]. Here, we have deployed a similar approach to purify a PI SVMP with a molecular weight of 23 kDa from the venom *C. atrox*. The mass spectrometry analysis of this purified protein (entitled CAMP-2) suggests that this is highly likely to be a previously sequenced protein, atroxase, from the same venom. Further functional assays confirmed that CAMP-2 is a collagenolytic, fibrinogenolytic and mildly haemolytic enzyme. At a relatively high concentration, CAMP-2 was also found to inhibit human platelet aggregation. These functions of CAMP-2 are very similar to the ones that have been reported for atroxase (a non-haemolytic protease with fibrin(ogen)olytic activity) [20,23]. Although the complete sequence of CAMP-2 was not obtained in this study, based on the mass spectrometry and functional data, this protein is highly similar, if not identical, to atroxase.

Given that SVMPs directly and indirectly mediate local tissue damage, the inhibition of these enzymes is likely to reduce SBE-induced local tissue damage [13]. Here, the sensitivity of CAMP-2 to MMP inhibitors (batimastat and marimastat) was tested in comparison to the whole *C. atrox* venom. There is a great degree of both structural and functional homology between SVMPs and human variants of MMPs [31]. This implies that substrate/inhibitor interactions between these subfamilies are likely to be similar [31]. Marimastat is a hydroxamic acid derivative which exerts broad-range metalloprotease inhibition by mimicking the cleavage site of collagen substrates [31]. When comparing the structural differences between these two compounds, marimastat has an additional hydroxyl group, increasing its hydrophilicity and thus, improving its pharmacokinetic properties [32]. Although they are likely to inhibit the majority of SVMPs, the additional domains found in PII, PIII, and PIV SVMPs would most likely be unaffected by these inhibitors. Indeed, batimastat and marimastat have already been reported to inhibit SVMPs in various venoms under in vitro and in vivo [33,34,35] settings. Similar to previous studies, the metalloprotease activity of both whole venom and CAMP-2 were almost completely inhibited by MMP inhibitors at around 3 μM. Notably, as a PI SVMP, CAMP-2 possesses only the metalloprotease domain. These data emphasise that batimastat and marimastat are both likely to act as broad-spectrum inhibitors for SVMPs including the PI SVMP analysed in this study. In contrast to these inhibitors, which target only the metalloprotease domain of SVMPs, antivenoms, on the other hand, are probably capable of binding all, or many of, the domains including the metalloprotease domain in SVMPs. While the large size of antibodies affects their ability to reach the bite site in time to prevent local tissue damage, the small molecule inhibitors are likely to reach and act rapidly for SBE-induced tissue damage. Multiple injections of these inhibitors have also been suggested to treat SBE, although the consequences of inhibiting human MMPs in the body are yet to be elucidated.

In the past, EDTA has been used to treat SBE under clinical/in vivo settings [36]. As a chelating agent, it binds to divalent cations and therefore affects the catalytic activity of metalloproteases. Several studies have reported the impact of EDTA on venom and other human metalloprotease activities [36]. In this study, EDTA has inhibited the metalloprotease activity of both the venom and CAMP-2; however, its use is problematic, and being orally bioavailable, marimastat is a far better clinical approach, especially considering the passing of stage II clinical trials. Similarly, ZnCl_2_ has proven to be a successful inhibitor of SVMPs through its ability to cause stereochemical and structural instabilities of metalloproteases when used in excess [37,38]. ZnCl_2_ has also shown a significant inhibitory effect on the metalloprotease activity of whole venom and CAMP-2 at many of the concentrations tested. Another observation is that the plateau of the SVMP activity observed at 25, 50 and 100 µM concentrations when the whole venom was used. This may be as a result of Zn^2+^ saturation, which may have caused Zn^2+^ to bind to other ions and proteins within the whole venom, rather than just the metalloproteases [39]. 

As marimastat and batimastat have proven to be effective in the inhibition of SVMP activity of CAMP-2, a cytotoxicity assay was completed to establish whether they are cytotoxic to human cells, such as platelets. As the LDH cytotoxicity assay measures whether the plasma membrane is damaged, this can be correlated with the effects that the compounds would have on other cells in the human body. If they present signs of cytotoxicity, they would not be appropriate for human use. However, both marimastat and batimastat showed no significant cytotoxic effects on platelets at the numerous concentrations tested and also passed phase I safety trials in humans [28]. This highlights their potential for further development into next-generation treatments for SBE in humans. However, the long-term side effects of their use and dosage requirements are still unknown and demand extensive in vivo research before they can be fully supported as an adjunctive treatment for SBE. The use of structural biology to screen and identify small molecules that are likely to affect venom toxins is also beneficial in finding alternative treatments for SBE. In this study, the structure of CAMP-2 was modeled and used in a docking analysis with batimastat and marimastat. Batimastat possessed higher binding energy and inhibitory constant, whereas marimastat has formed multiple hydrogen bonds with the active site of CAMP-2. While batimastat was found to interact with one of the catalytic triad histidine (His 154) residues through its backbone nitrogen, marimastat was observed to form a bifurcated hydrogen bond interaction with another active site residue, glutamate (Glu 145), through its sidechain oxygen. Due to the easy access and robustness of these *in silico* approaches, they can be used to analyse the binding efficiencies of these and other similar molecules with SVMPs from diverse venoms to determine their potential in treating SBE.

SVMPs play important roles in the overall pathophysiology of viper envenoming by inducing local tissue damage and haemorrhage, which can be primarily attributed to their potential to degrade basement membrane components and affect coagulation factors [18,40]. SVMPs activate two key coagulation factors, factor X and prothrombin, to exhibit their procoagulant effects. SVMPs are also known to digest plasma fibrinogen, which may induce clotting effects in some cases, but, largely, it induces the consumption coagulopathy by reducing the functional fibrinogen levels in the plasma, which subsequently results in bleeding. CAMP-2 has also exerted its ability to degrade fibrinogen, specifically the Aα chain, with delayed/minimal effect on Bβ chain. SVMPs [18,40], as well as venom serine proteases [41], show varying specificity to cleave the Aα and/or Bβ chains of fibrinogen with rarely an effect on the γ chain. Therefore, in addition to their impact on local tissue damage, the effects of SVMPs on the blood coagulation factors, specifically fibrinogen, should also be reduced in order to combat SBE-induced pathological complications.

In conclusion, SVMPs are one of the key venom toxins that should be neutralised as quickly as possible following a snakebite, particularly in the case of vipers. The inability of antivenoms to counteract the effects of SVMPs, particularly at the local bite site, emphasises the urgent need to develop alternative, small molecule-based treatments to minimise SBE-induced tissue damage, which often results in permanent disabilities. In this study, we give a comprehensive analysis of the applications of batimastat, marimastat, EDTA and ZnCl_2_ in inhibiting a purified PI SVMP, CAMP-2, in comparison to the whole *C. atrox* venom. The cytotoxic effects of batimastat and marimastat were also analysed. Hence, this study demonstrates a robust method to screen small molecule inhibitors for therapeutic utility against venom toxins using a spectrum of functional assays and *in silico* techniques in order to develop alternative therapies for SBE.

## 4. Materials and Methods

### 4.1. Protein Purification

Lyophilised *C. atrox* venom (50 mg) (Sigma Aldrich, Dorset, UK) was dissolved in 1 mL of 20 mM Tris.HCl (pH 7.4) (ThermoScientific, Loughborough, UK), and after centrifugation at 5000× *g* for 5 min to remove the undissolved materials, the supernatant was applied to a 5-mL HiTrap™ Sepharose (SP) HP cation exchange chromatography column (GE Healthcare, Amersham, UK) and fractionated using an Akta Purifier (GE Healthcare, Amersham, UK). Fractions were collected at a rate of 1 mL/min using 20 mM Tris.HCl pH 7.4 (Buffer A) and 1 M NaCl prepared in Buffer A (Buffer B) with a gradient reaching 60% Buffer B over 30 min. All fractions containing the target protein were pooled together, desalted and concentrated using Vivaspin ultra-centrifugal filtration tubes (Sartorius, Epsom, UK), and applied to a gel filtration column (Superdex 75) to further purify the target protein. Fractions were collected at a rate of 1 mL/min using 20 mM Tris.HCl (pH 7.4).

The fractions were stored on ice and analysed by Bradford protein assay according to the manufacturer’s protocol (ThermoScientific, Loughborough, UK) and SDS-PAGE, as described previously [42]. The protein assay was run, and the intensity of the color developed was measured at 600 nm using an Emax spectrophotometer (Molecular Devices, Wokingham, UK). Bovine serum albumin (BSA) (ThermoScientific, Loughborough, UK) was used as a standard in the protein assay. For SDS-PAGE, the proteins were denatured using reducing sample treatment buffer (RSTB) (10% (*w*/*v*) SDS (ThermoScientific, Loughborough, UK), 10% (*v*/*v*) β-mercaptoethanol (Sigma Aldrich, Dorset, UK), 1% (*w*/*v*) bromophenol blue (Sigma Aldrich, Dorset, UK), 50% (*v*/*v*) glycerol (ThermoScientific, Loughborough, UK) and 20 mM Tris.HCl (pH 7.4)) and heating at 90 °C for 10 min. Samples (30 μL from each fraction) were loaded into precast gradient (4–15%) Mini-PROTEAN^®^ TGX™ gels (Bio-Rad, Watford, UK) alongside a protein molecular weight marker (Bio-Rad, Watford, UK) and resolved using a Mini-Protean II apparatus (Biorad, Watford, UK). Gels were immersed in a staining solution (0.1% (*w/v*) Coomassie Brilliant blue R250 (Sigma Aldrich, Dorset, UK) dissolved in 10% (*v*/*v*) acetic acid (ThermoScientific, Loughborough, UK), 40% (*v*/*v*) methanol (ThermoScientific, Loughborough, UK) and 50% deionized water) for 1 h on a plate shaker, washed 3x with deionised water for 5 min and destained (10% (*v*/*v*) acetic acid, 10% (*v*/*v*) methanol and 80% deionized water) for 3 h or until protein bands became clear. 

### 4.2. Mass Spectrometry Analysis

A gel slice (from SDS-PAGE) containing CAMP-2 was subjected to tryptic digestion before undergoing mass spectrometry analysis at Alta Bioscience (Birmingham, UK), as we described previously [14]. Both MS and MS/MS scans were cross-referenced against the Uniprot protein database using the Sequest algorithm (Thermo fisher PD 1.4) in order to determine the identity of the purified protein.

### 4.3. Fluorogenic Assays

The metalloprotease (collagenolytic) activity of the venom or the purified protein (CAMP-2) was measured using a fluorogenic substrate, DQ™-gelatin (ThermoScientific, Loughborough, UK). Several concentrations of the purified protein or venom (with and without different concentrations of batimastat, marimastat, EDTA, ZnCl_2_ and CaCl_2_ (Sigma Aldrich, Dorset, UK)) were added to a black 96-well plate in triplicates along with appropriate controls. Then DQ-gelatin (10 μg/mL) was added to each well, and following mixing, the plate was incubated at 37 °C and the level of fluorescence was measured at various time points using an excitation of 485 nm and emission wavelength of 520 nm in a FLUOstar OPTIMA (BMG Labtech, Ortenberg, Germany) spectrofluorimeter. To determine PLA_2_ activity, an EnzChek™ Phospholipase A_2_ Assay Kit (ThermoFisher Scientific, Loughborough, UK) was used in accordance with the manufacturer’s instructions. 

### 4.4. Fibrinogenolytic Assay

CAMP-2 (1 mg/mL) was mixed with fibrinogen (10 mg/mL) (Sigma Aldrich, Dorset, UK) in PBS and incubated at 37 °C for various time points. Samples (50 μL) were taken at time intervals of 10, 30 and 60 min and then again after 12 h and immediately mixed with 25 μL of RSTB before boiling at 90 °C for 10 min. Each sample was then analysed by SDS-PAGE, as explained above. 

### 4.5. Human Blood Collection, Platelet Preparation and Aggregation Assay

Blood samples from healthy human volunteers were obtained in accordance with the approved procedures by the University of Reading Research Ethics Committee (UREC 17/17 approved: 10 May 2017) and after obtaining written informed consent. The platelets were prepared, as described previously [42]. Blood was collected using venepuncture into vacutainers containing 3.2% (*w*/*v*) citrate. For PRP, blood samples were centrifuged at 102× *g* for 20 min at 20 °C. PRP was rested for 30 min at 30 °C in a water bath before use. For isolated platelets preparation, the PRP was mixed with 3 mL of acid citrate dextrose (ACD) and 10 ng/mL prostacyclin (PG1_2_ dissolved in EthOH) (Sigma Aldrich, Dorset, UK) and mixed gently by inversion and centrifuged at 1413 *g*. Modified tyrodes-HEPES buffer (1 ml with 5 mM glucose) was added together with 150 μL of ACD to the platelet pellet to resuspend the pellet, and the final volume was made up to 25 mL using a pre-warmed modified tyrodes-HEPES buffer. A further 3 mL of ACD and 10 ng/mL prostacyclin was added prior to centrifuging at 1413× *g* for 10 min at 20 °C. Finally, the supernatant was discarded, and the platelets were resuspended in modified tyrodes-HEPES buffer at a density of 4 × 10^8^ platelets/mL. The aggregation assays were performed using isolated platelets or PRP with 0.5 μg/mL CRP-XL (obtained from Professor Richard Farndale, University of Cambridge, UK) as an agonist. The level of aggregation in the presence and absence of different concentrations of venom or CAMP-2 was monitored using an optical aggregometer (model 700, Chrono-log, USA). 

### 4.6. Haemolytic Assay

Human erythrocytes were collected from the dense red blood cells found in the bottom of vacutainers following centrifugation for the collection of PRP, as mentioned above. These erythrocytes were then washed three times by mixing with an equal volume of PBS, centrifuging at 2000× *g* for 2 min, and discarding the supernatant. The haemolytic activity was measured using these washed human erythrocytes suspended in calcified phosphate-buffered saline (PBS). The erythrocytes were treated with different concentrations of CAMP-2 or venom and incubated at 37 °C for various time points. A detergent, Triton X-100 (1%) (Sigma Aldrich, Dorset, UK), and PBS were used as a positive and negative control, respectively. Following incubation, the samples were centrifuged at 2000× *g* for two minutes and 50 μL of supernatant was pipetted into a 96-well plate and the absorbance was measured at 540 nm using a spectrophotometer.

### 4.7. LDH Cytotoxicity Assay

An LDH cytotoxicity assay kit (ThermoFisher, Loughborough, UK) was used in accordance with the manufacturer’s instructions. Briefly, human platelets were incubated at 37 °C for 30 min prior to incubation with different concentrations of venom or CAMP-2 or small molecule inhibitors for five minutes. These results were compared with those from the positive control (100% lysis) achieved using the lysis buffer provided. The substrate mix from the kit was added to the platelets and incubated for another 30 min and subsequently stopped using the stop solution provided. The level of absorbance was read at 490 and 650 nm using a Fluostar Optima (BMG Labtech, Ortenberg, Germany) spectrofluorimeter. The assay was performed in duplicates using platelets obtained from three individual donors.

### 4.8. Structure Modelling and Molecular Docking

A three-dimensional structure of CAMP-2 was developed using homology modeling in the Swiss Model Server [24] based on the crystal structure of adamalysin II (PDB accession number: 4AIG) from the venom of *Crotalus adamanteus* as a template. Before proceeding to the docking simulation, protein preparation steps such as fixing charge for the Zn^2+^ metal ion, adding solvation parameters and polar hydrogens to the metalloprotease were carried out. AutoDock [25] necessitates pre-calculated grid maps for each type of atom present in the ligand molecule being docked because it stores the potential energy produced from interacting with the macromolecule. This grid surrounds the region of interest (active site) of the macromolecule. A grid box of size 50 × 50 × 50 Å with a spacing of 0.375 Å was prepared at the active site of CAMP-2 metalloprotease. The Lamarckian genetic algorithm was used to identify the best conformers. Throughout the docking process, a maximum of 15 conformers was considered per compound. AutoDock (version 4.0 SRC, California, USA) was compiled and run under Microsoft Windows XP operating system.

### 4.9. Statistical Analysis

All statistical analyses were performed with GraphPad Prism (version 7.0, Graphpad Prism, California, USA, 2018). For most of the data, the statistical significance was analysed using one-way ANOVA, which was followed by a posthoc Tukey’s test.

## Figures and Tables

**Table 1:** Results of molecular docking of CAMP-2 with the inhibitors batimastat and marimastat using AutoDock (a molecular docking tool).

Compound	Binding Energy (kcal/mol)	Ligand Efficiency (kcal/mol)	Inhibitory Constant (μM)	H Bond Interactions (D-H…A)	Distance (Å)
Batimastat	−5.59	−0.17	79.37	ALA 114 N-H…O HIS 154 N-H…O	2.8 2.8
Marimastat	−5.29	−0.23	132.52	N-H…O LYS 109 ILE 111 N-H…O GLY 112 N-H…O N-H…O(E2) GLU 145 O-H…O(E2) GLU 145	3.2 3.2 3.0 2.6 3.1
